# MR image reconstruction from undersampled data for image-guided radiation therapy using a patient-specific deep manifold image prior

**DOI:** 10.3389/fonc.2022.1013783

**Published:** 2022-11-21

**Authors:** Jace Grandinetti, Yin Gao, Yesenia Gonzalez, Jie Deng, Chenyang Shen, Xun Jia

**Affiliations:** Innovative Technology of Radiotherapy Computations and Hardware (iTORCH) Laboratory, Department of Radiation Oncology, University of Texas Southwestern Medical Center, Dallas, TX, United States

**Keywords:** MRI, image reconstruction, radiotherapy, image guidance, prior information, patient-specific prior, deep learning, interpretable

## Abstract

**Introduction:**

Recent advancements in radiotherapy (RT) have allowed for the integration of a Magnetic Resonance (MR) imaging scanner with a medical linear accelerator to use MR images for image guidance to position tumors against the treatment beam. Undersampling in MR acquisition is desired to accelerate the imaging process, but unavoidably deteriorates the reconstructed image quality. In RT, a high-quality MR image of a patient is available for treatment planning. In light of this unique clinical scenario, we proposed to exploit the patient-specific image prior to facilitate high-quality MR image reconstruction.

**Methods:**

Utilizing the planning MR image, we established a deep auto-encoder to form a manifold of image patches of the patient. The trained manifold was then incorporated as a regularization to restore MR images of the same patient from undersampled data. We performed a simulation study using a patient case, a real patient study with three liver cancer patient cases, and a phantom experimental study using data acquired on an in-house small animal MR scanner. We compared the performance of the proposed method with those of the Fourier transform method, a tight-frame based Compressive Sensing method, and a deep learning method with a patient-generic manifold as the image prior.

**Results:**

In the simulation study with 12.5% radial undersampling and 15% increase in noise, our method improved peak-signal-to-noise ratio by 4.46dB and structural similarity index measure by 28% compared to the patient-generic manifold method. In the experimental study, our method outperformed others by producing reconstructions of visually improved image quality.

## 1 Introduction

### 1.1. MR guided radiation therapy

Online image guidance plays an important role for the success of radiation therapy (1). Using an imaging device installed on the medical linear accelerator (LINAC) to acquire patient anatomy images immediately before, or during treatment delivery, online image guidance allows the visualization of the patient anatomy at the treatment stage, which is essential to align the tumor target accurately with the radiation beam, or to adjust the treatment plan based on the anatomy to maintain the plan optimality. Imaging during the treatment delivery also permits motion monitoring of the tumor and other organs to ensure delivery accuracy and patient safety. Cone beam CT achieved using an x-ray tube and a flat panel detector mounted on a LINAC is currently the most widely used image guidance modality (2). Nonetheless, its applications are impeded by the low soft-tissue contrast, the associated ionizing radiation, and the lack of real-time imaging capabilities. Lately, technological advancements have enabled the integration of a Magnetic Resonance (MR) imaging scanner with a LINAC, yielding the new scheme of MR-guided radiation therapy (MRgRT) (3). Adoption of this novel scheme has demonstrated improved tumor targeting accuracy, allowing increased radiation dose to the tumor, reduced dose to nearby critical organs, and therefore better treatment outcomes (4).

### 1.2. MR reconstruction with undersampled data

For online image-guidance purposes, fast data acquisition is needed to speed up the overall imaging process. Otherwise, motion of patient anatomy may occur during the image acquisition process, defeating the purpose of image-guidance. Hence, in MRgRT, MR image reconstruction with undersampled data is of particular importance. This problem has been extensively studied over the years. Generally speaking, additional information has to be provided to the reconstruction workflow to compensate the missing information from the undersampled data. This can be often achieved by introducing regularization on image quality. For instance, Tikhonov regularization was invented to ensure overall smoothness of the solution (5). In the Compressive Sensing (CS) framework (6–8), an optimization problem was formed with the regularization term included in the objective function. The key ingredient was to find a transformation, such that the unknown image to be restored is sparse under the transformation. Enforcing the sparsity of the solution can be used *a priori* as a regularization in the optimization problem by minimizing the *L*_0_ norm of the transformed image, which is often relaxed into minimizing the *L*_1_ norm because of better numerical properties. Under this framework, a number of transformations have been explored in different image processing problems. The popular Total variation (9, 10) method used a gradient transform to ensure the solution sparsity under this transform. Wavelet (11) and similar transforms, such as tight wavelet frame (12, 13), were employed to regularize the solution image at multiple resolutions with the wavelet basis functions. The idea of low-rank regularization was also introduced (14, 15). In the problem of reconstructing images with motions, the matrix composed of images at multiple time points as columns naturally has a low rank due to the similarity among images. This property was introduced into the reconstruction problem by penalizing the rank. In an effort to find basis functions that can effectively sparsify an image, a dictionary learning approach was developed, which learns a dictionary basis from existing MR images. The learned dictionary was then used in image reconstruction to regularize the unknown image by requiring that it has a sparse representation under the basis (16).

Recently, deep learning (DL) has demonstrated its power in solving a number of machine learning problems in different domains (17–20). With a deep neural network (DNN) of a large-scale hierarchical multi-layer structure, it is possible to approximately represent a very complex distribution of the dataset of interest. When applying to MRI image processing problems, this enables description of the desired image properties, which is valuable to enforce solution quality. A straightforward approach is to use a DNN to map a low-quality MR image to a high-quality image. Kwon et al. employed a multi-layer perceptron (21) and Lee et al. used a deep residual network (22) to reduce aliasing artifact of MR images caused by data undersampling. Chun et al. developed a DNN to map a low-resolution MR image to the high-resolution counterpart, achieving the goal of super-resolution (23). In the k-space data domain, a DNN was used to interpolate data to address the missing data problem in the undersampling situation to improve the quality of reconstructed images (24). Lately, feasibility of training a DNN to map data directly from k-space to the image space has also been demonstrated, hence achieving the MR image reconstruction task bypassing the physics-based reconstruction process (25). Viewing the iterative reconstruction process as the data processing pipeline in a feed-forward DNN, it was proposed that we could learn parameters in the iterative process, such as image filter kernels, activation functions, and weighting factors *via* the network training process. This idea was realized for a few commonly used iterative MR image reconstruction algorithms including the gradient descent algorithm (26) and the alternating direction method of multipliers (ADMM) (27). Sriram et al. introduced a novel End-2-End Variational network to train the network in an end-to-end fashion for multi-coil fast MRI reconstruction (28).

### 1.3. Manifold constrained image reconstruction

Generally speaking, when dealing with undersampled data, effectiveness of a reconstruction algorithm lies in how effectively it can provide prior information to compensate missing measurements or errors in the data. Mathematically, the prior information forms a low-dimensional manifold with an intrinsic dimension much lower than the high dimensional image space. Images on the manifold follow the characteristics of the desired images, such as their appearance, intensity, structural contents, etc. The manifold can thus serve as a constraint to the solution image to ensure its desired characteristics. This idea has been explored in a number of studies using different approaches to construct the manifold and utilize it. In a seminal work, Chen et al. used a Compressive sensing approach to constrain the solution similar to a prior image (29). Researchers successfully built manifolds by explicitly modeling the data structure, e.g. using a kernel-based method (30), manifold learning (31, 32), and analysis dictionary learning and manifold structure regularization (ADMS) (33). The manifolds were then incorporated in the problems of MR image reconstruction (34, 35) or MR parameter mapping (32). With the flexibility of using DNNs to represent a manifold, DL allowed the modeling of the image manifold *via* the network training process, as well as the incorporation of the manifold in image reconstruction. The power of this approach has been demonstrated in both MR and CT reconstruction problems (36–38).

Along this line, when constructing the manifold from prior image data, the effectiveness of the manifold in the subsequent image reconstruction problem depends on the relevance of the prior image data to the image to be reconstructed. Previous studies (29, 34–38) generally used *patient-generic* prior images to construct the manifold, because these images are widely available and the manifold can effectively provide prior information such as image intensity, structure, etc, to help the reconstruction task. Nonetheless, it is expected that the effectiveness of the manifold can be further enhanced by a *patient-specific* manifold, i.e. using images of a patient to construct the manifold and use it to reconstruct images for the same patient. This approach may further provide patient-specific information to the image reconstruction process.

### 1.4. Our contributions

One of the contexts enabling the use of this patient-specific prior information is MRgRT. In radiotherapy, high-quality *patient-specific* MR images are available for treatment planning purposes. Hence, the image can be naturally used to build the manifold specifically for this patient. At the treatment stage, when a new MR image is to be reconstructed to guide the treatment delivery, the manifold can be used to facilitate the reconstruction task. This may allow rapid data acquisition with undersampling, while delivering high-quality reconstructed MR images. In this paper, we present our recent study on MR image reconstruction for MRgRT using the patient-specific image manifold prior constructed *via* DL. The major contributions of this work are twofold.

1. We point out that the clinical context of MRgRT permits the use of patient-specific prior information to aid the reconstruction of MR images for image-guidance purposes. It is innovative to notice this property that leads to more effective modeling of prior information than using the patient-generic approach.
2. We develop a DNN to learn the patient-specific image manifold prior, as well as a reconstruction algorithm to incorporate the manifold to the reconstruction process. We demonstrate the effectiveness of this approach and the superior performance over other reconstruction methods, including the one with a patient-generic manifold. The manifold-based reconstruction algorithm also makes the image restoration process interpretable, facilitating the clinical application.

The remaining sections are organized as following: Section 2 will present our method including manifold construction, its incorporation in reconstruction, and additional implementation details. In Section 3, we will conduct extensive tests to evaluate the proposed method and demonstrate its effectiveness. After giving discussions in Section 4, we will conclude this work in Section 5.

## 2 Materials and methods

**Figure 1:**
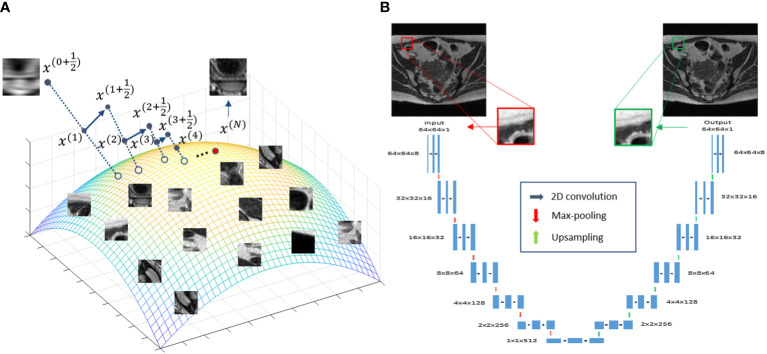
**(A)** Illustration of the prior information manifold and the reconstruction process. **(B)** Architecture of the DNN used to represent the manifold.

The overall idea of the proposed approach is illustrated in **Figure 1A**. The colored surface represents a low-dimension manifold of the prior information to be constructed from patient-specific prior images. Once the manifold is learned from the data, it serves as a constraint during the iterative reconstruction process, such that the solution resides on this manifold.

### 2.1. Deep neural network-based patient specific image prior

In this study, we used a DNN to represent the manifold formed by patches extracted from the patient’s prior MR image available at the radiotherapy treatment planning stage. We considered the manifold of image patches, because in MRgRT there is often only one image available for treatment planning. Breaking an image into a number of patches provided sufficient data for model training.

To represent the patch-based manifold, we used a deep auto-encoder (DAE) architecture (39). A DAE first maps input data to a low-dimensional latent space, based on which it restores the input data exactly. This dimension reduction established by the DAE is required to preserve all useful information for exact data recovery. As such, a DAE is a natural choice for representing a manifold, and has been widely employed in previous studies (38, 40–45). Here, we built a DAE Φ(·|*θ*)=*D*(*E*(·|*θ*_*E*_)|*θ*_*D*_) with its detailed architecture shown in **Figure 1B**, where *D*(·|*θ*_*D*_) and *E*(·|*θ*_*E*_) are the decoder and encoders, respectively. *θ*_*D*_ and *θ*_*E*_ are network parameters. Φ(·|*θ*) denotes the overall mapping with *θ*={ *θ*_*D*_,*θ*_*E*_} . The DAE took a patch *x*_*i*_,(*i*∈Ω) of size 64×64 centered at the *i* -th pixel of the prior MR image as input, and first mapped it to a low-dimensional latent space *via* the first half (encoder) of the DAE, i.e. *z*_*i*_=*E*(*x*_*i*_|*θ*_*E*_) . In this study, *z*_*i*_∈*R* ^512^ indicating that the dimension of the input patch was reduced by a factor of 8 *via* the encoder. The latent representation *z*_*i*_ was then fed into the second half of the network (decoder) to recover the input, i.e. *x*_*i*_=*D*(*z*_*i*_|*θ*_*D*_) .

Training the DAE was formulated as an optimization problem, enforcing the DAE to exactly recover input image patches


(1) $$\hat{\theta }=\arg\min_{\theta }\sum_{i\in \Omega }L(\Phi (x_{i}|\theta ),x_{i})$$

where the loss function *L*(·) can be any metric measuring distance between Φ(*x*_*i*_|*θ*) and *x*_*i*_ . We used the mean absolute error (MAE) as the metric in this study. This optimization problem was solved iteratively *via* the gradient descent algorithm, i.e.


(2) $$\theta^{\left(t\right)}=\theta^{\left(t-1\right)}-\delta \nabla_{\phi }L\frac{\partial \Phi }{\partial \theta }{|}_{\theta =\theta^{\left(t-1\right)}},t=1,2,\ldots$$

where *δ* is a constant specifying the step size in each update, and is often referred as learning rate in the DL regime. $\frac{\partial \Phi }{\partial \theta }$ is evaluated in a layer-by-layer fashion based on the chain rule. Due to the large number of training samples in the training dataset, a stochastic gradient descent (SGD) algorithm was employed to improve the training efficiency, which evaluated the gradient using a random subset of the training data in each iteration. Training was performed until a maximal number of iterations was reached, while the optimal parameters denoted as $\hat{\theta }$ achieving the best performance was saved to generate the trained DAE $\text{Φ}(\cdot |\hat{\theta })$ to be used in the reconstruction step.

### 2.2. Reconstruction method

To incorporate the trained DAE as prior information, we formulated the following reconstruction model:


(3) $$\underset{x}{\min}∥FSx-g{∥}_{F}^{2}+\beta \sum_{i\in \Omega }∥P_{i}x-\Phi (P_{i}x|\hat{\theta }){∥}_{F}^{2}.$$

*x* represents the MR image to be reconstructed, and *g* denotes the acquired signal in k space. *F* indicates the 2D Fourier transform operator, while *S* is the undersampling operator. || . ||_*F*_ denotes Frobenius norm. *P*_*i*_ refers to the operator that extracts a 64-by-64 pixel image patch centered at the *i* -th pixel from *x* Ω is the set of all pixels in the image. The first term in Eq. (3) enforced the fidelity between the reconstructed image and the acquired k-space data. The second term incorporated the trained DAE $\text{Φ}(\cdot |\hat{\theta })$ to regularize the quality of the reconstructed image. *β* is a regularization parameter balancing the contributions from the two terms. By solving this optimization problem, we expect to reconstruct an MR image of similar image quality as the prior image, while its content is defined by the measurement *g* , reflecting the anatomy/structure at the time of scanning instead of being biased towards the prior information.

To solve Eq. (3), we employed the forward-backward splitting algorithm. More specifically, the original problem was solved by tackling the following two subproblems alternatively in each iteration until convergence:


$$x^{\left(t+\frac{1}{2}\right)}={\text{argmin}}_{x}║FSx-g{║}_{F}^{2},$$

(4) $$x^{\left(t+1\right)}=\arg\min_{x}\beta \sum_{i\in \Omega }∥P_{i}x-\Phi (P_{i}x|\hat{\theta }){∥}_{F}^{2}+∥x-x^{\left(t+\frac{1}{2}\right)}{∥}_{F}^{2}.$$

The first subproblem is of a quadratic form, which can be solved efficiently using the conjugate gradient least-square algorithm (46) with the initial guess set as *x* ^(*t*)^ , i.e. the solution obtained in the previous iteration step. In this study, we assumed the solution is a real image and hence enforced this at this step of the iterative process by taking the absolute value.

The second subproblem in Eq (4), on the other hand, involves the DAE, which is a highly non-linear and non-convex function. To solve such a complex optimization problem, we proposed to employ a fix-point scheme, i.e. at each iteration, we fix $\text{Φ}(P_{i}x|\hat{\theta })$ as $\text{Φ}(P_{i}x^{\left(t+\frac{1}{2}\right)}|\hat{\theta })$ and hence the second subproblem becomes


(5) $$\arg\min_{x}\beta \sum_{i\in \Omega }∥P_{i}x-\Phi (P_{i}x^{\left(t+\frac{1}{2}\right)}|\hat{\theta }){∥}_{F}^{2}+∥x-x^{\left(t+\frac{1}{2}\right)}{∥}_{F}^{2}.$$

The solution to this modified optimization problem can be obtained explicitly as


(6) $$x^{\left(t+1\right)}=\frac{\beta \sum_{i\in \Omega }P_{i}^{*}\Phi (P_{i}x^{\left(t+\frac{1}{2}\right)}|\hat{\theta })+x^{\left(t+\frac{1}{2}\right)}}{\beta n^{2}+1},$$

where $P_{i}^{*}$ is the adjoint operator of *P*_*i*_ , which simply places the extracted patch *i* back to its location in the MR image. In this case, $P_{i}^{*}P_{i}x=n^{2}x$ , where *n* is the patch size. Note that we used one patch for each pixel, so the patches were overlapping and Each pixel was covered by *n* ^2^ patches. In the above equation, the summation indicated that the adjoint operator added each patch to corresponding locations, and overlapping patches were summed. The normalization was reflected in the denominator term of this equation. In general, if patches were overlapped, the updated pixel value would be the average over all patches covering this pixel, together with the $x^{\left(t+\frac{1}{2}\right)}$ term.

The complete update scheme to solve the reconstruction problem in 3 is summarized in **Algorithm 1**.

Algorithm 1 DAE assisted reconstruction algorithm. Input: *x*
^(0)^ *F, S, g, P*
_
*i*
_


$\text{Φ}\left(\cdot |\hat{\theta }\right)$


*β* *∈*
_0_, *t*
_
*max*
_
Output:


$\hat{x}$

Initialization: *set*
^
*t*=0^
Step 1: compute x


$\left(t+\frac{1}{2})=ConjugateGradient(x^{\left(t\right)},F,S,g\right)$
Step 2: Compute x 
$(t+1)=\frac{\beta {\sum_{i\in \Omega }P}_{i}^{*}\Phi (P_{i}x^{\left(t+\frac{1}{2}\right)}|\hat{\theta })+x^{\left(t+\frac{1}{2}\right)}}{\beta n^{2}+1}.$
Step 3: if *t*<*t*
_
*max*
_ and 
$\frac{∥x^{\left(t+1\right)}-x^{\left(t\right)}{∥}_{F}^{2}}{∥x^{\left(t\right)}{∥}_{F}^{2}}>\in_{0}$
 set t=t+1, go to Step 2
Step 4: Set 
$\hat{x}=x^{\left(t\right)}$

The interpretability of the reconstruction **Algorithm 1** can be illustrated in **Figure 1A**, which offers a geometric visualization about the iterative reconstruction process. At the beginning of each iteration, with the solution *x* ^(*t*)^ as the initial guess, solving the least square problem in step 1 generates a new solution $x^{\left(t+\frac{1}{2}\right)}$ . For each patch $x_{i}=P_{i}x^{\left(t+\frac{1}{2}\right)}$ , the DAE generates a corresponding one on the manifold by first computing the coordinate in the latent space $E(x_{i}|{\hat{\theta }}_{E})$ and then restoring the patch for this coordinate ${\hat{x}}_{i}=D(E(x_{i}|{\hat{\theta }}_{E})|{\hat{\theta }}_{D})$ . These patches are assembled to form a new image. Finally, the new image and $x^{\left(t+\frac{1}{2}\right)}$ are averaged with weights proportional to *β* and 1 , respectively, yielding a new solution *x* ^(*t*+1)^ . This process continued until convergence, where a solution on, or sufficiently close to, the manifold prior and meeting the data fidelity was generated.

### 2.3. Implementation details

The proposed training and reconstruction framework was implemented using Python with TensorFlow (47) on a sever equipped with eight Intel Xeon 3.5 GHz CPU processors, 32 GB memory and one Nvidia V100 GPU card. In our experiment, all the images were first normalized to [ 0,1 ] for simplicity. The DAE was trained with a learning rate of 1×10^−5^ and a batch size of 64. The maximal training epoch number was set to 10,000, while the training will be terminated if the average MAE was lower than 1×10^−5^, or no further improvement was observed in 100 epochs. The time to train the DAE was about 2 days.

As for the reconstruction using the trained DAE, the maximal number of iterations was set to *t*_*max*_=15 , while the algorithm would be stopped earlier if the relative difference between the solutions of two consecutive iterations was less than *∈*_0_=10^−3^ . We manually adjusted the value of the parameter *β* . The best value was determined based on the resulting image quality.

### 2.4. Evaluation studies

#### 2.4.1. Simulation study

**Figure 2:**
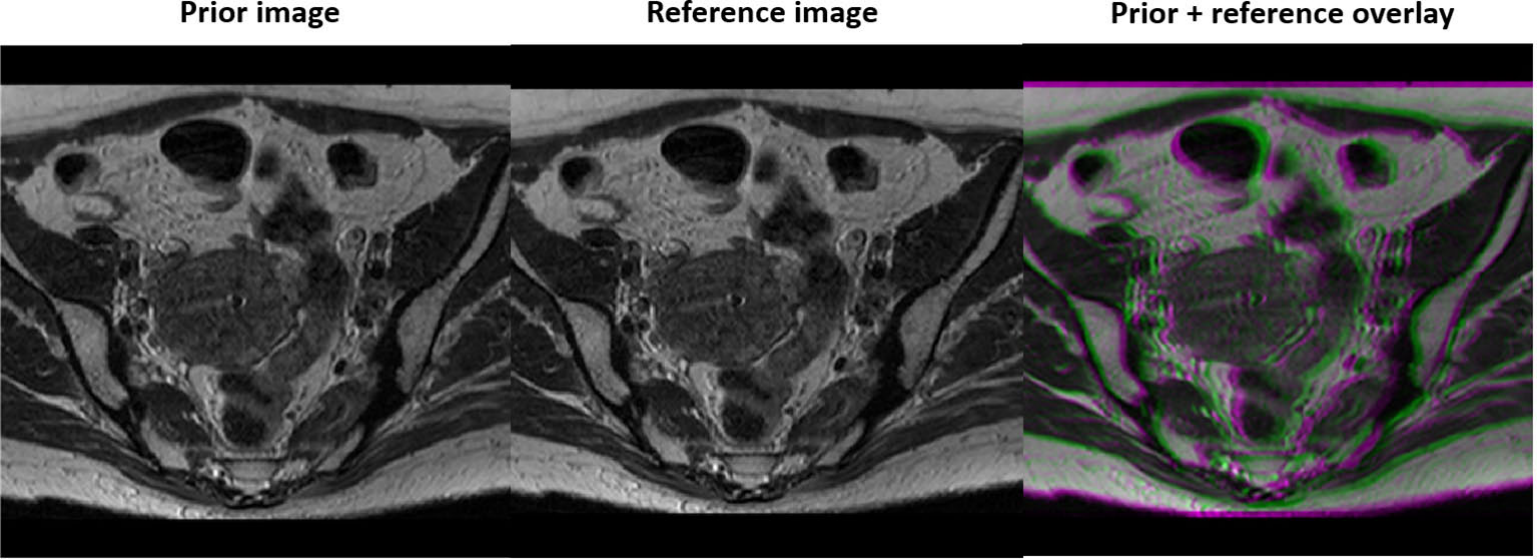
Prior MR image (left), MR image with simulated motion (middle), and the overlay of the two illustrating deformation between the two (right).

We first evaluated the performance of the trained DAE in terms of recovering the input image patches, and as prior information in reconstruction in a simulation study using an abdominal MR image of a patient treated at our institution. We followed the standard simulation protocol of gynecological cancer in our clinic to acquire images using a 3D T2W turbo spin echo sequence. The parameters for this sequence included Repetition Time (TR) 1250 ms, Time to Echo (TE) 185 ms and flip angle 70 degree. The MRI image was acquired for radiotherapy treatment planning with a resolution of 512×512 voxels and a voxel size of 0.71 mm. One image patch was extracted for each pixel in the image, resulting in 262,144 training samples to train the DAE. During a radiotherapy treatment course, patient anatomy varies from day to day but is expected to be similar to the prior image up to a deformation. Hence, we deformed the prior image with a smooth motion vector field with maximum amplitude of 14.2 mm to generate the test image to reconstruct, see **Figure 2**.

We first trained the DAE with the patches extracted from the prior image. To examine the quality of the DAE, we fed the DAE with image patches extracted from both the training and testing images. The output from the DAE was compared against the input both visually and quantitatively to evaluate the quality of the training process.

**Figure 3:**
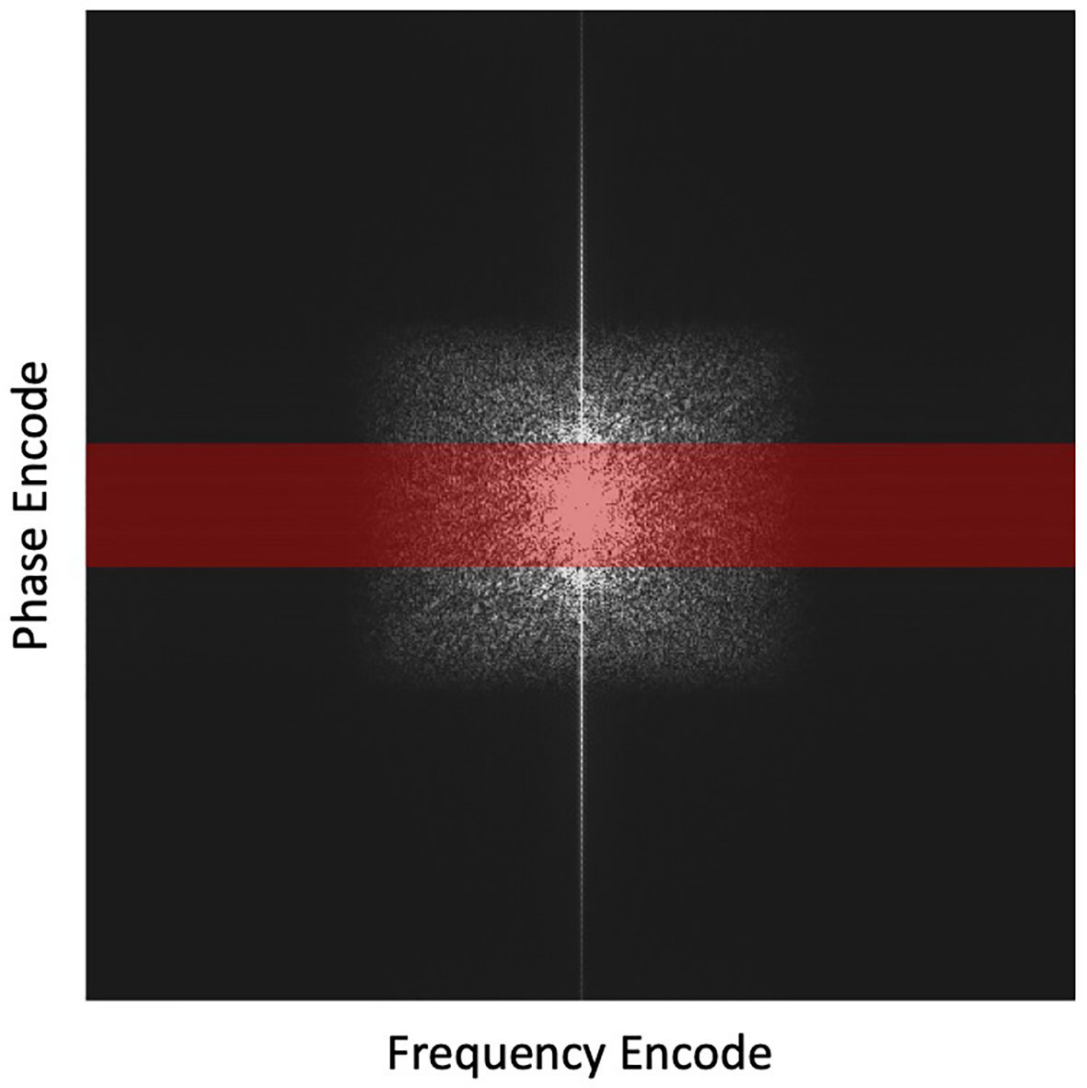
Highlighted region in red depicts the undersampling scheme with the frequency encoding =512 points and phase encoding reduced from 512 to 64 lines (by 8 times). This undersampling scheme is overlaid on a fully sampled k-space for illustration purposes.

After that, we incorporated the trained DAE into the proposed reconstruction scheme and investigated the reconstructed image quality. Full k-space data was obtained for the test image *via* Fourier transform of the image. Sampling of k-space was performed to keep the central lines of k-space, preserving the low frequency and high contrast components of the image. We reduced the number of phase encode lines, while fully sampling in the frequency encoding direction as no time penalty is incurred. The undersampling ratio was defined as the percentage of phase encode lines sampled. **Figure 3** depicts the undersampling scheme. With this scheme, it was hoped that the prior manifold trained with image patches would compensate the missing high-frequency information along the phase encoding direction. For all testing cases, we renormalized the intensity range of reconstructed image during the reconstruction process to [0,1]. Based on our experience, the signal intensity was not noticeably affected, as the down sampling ratio was changed, which was ascribed to the retention of the lower frequency components in our sampling scheme. Noise signals sampled from a Gaussian distribution of different amplitudes were added to the k-space data, mimicking the amplified noise due to reduced signal averaging to speed up the acquisition. It is desired that the reconstruction algorithm only utilizes the prior information to improve image quality, intensity accuracy and noise reduction, while preserving the anatomical structure from being biased by the prior image. We examined the resulting image quality visually from this perspective. Quantitatively, the image quality was measured using Structural Similarity Index Measure (SSIM) (48) and Peak Signal-to-Noise Ratio (PSNR).

To benchmark the proposed scheme, we compared results with those generated by three other reconstruction methods.

• The first was the direct Fourier Transform (FT) method, which is widely used in the standard clinical practice.

• The second method was a tight-frame (49) based CS reconstruction algorithm, which reconstructs the image by solving the following problem:


(7) $$\underset{x}{\min}∥FSx-g{∥}_{F}^{2}+\lambda ∥Wx{∥}_{1}.$$

• *W* is a set of tight frames. *λ* is the regularization parameter that was adjusted manually for the best image quality. The problem was solved using the Alternating Direction Method of Multipliers (50).

• As the main contribution of this study is the use of a patient-specific manifold, in the third comparison, we studied the algorithm with a patient-generic manifold as the constraint on image quality. The reconstruction algorithm followed exactly the same scheme as the proposed algorithm except that the DAE was trained to represent a general manifold for different patients. We collected abdominal MR images from another 14 patients and used patches randomly extracted from these images to train the patient-genetic DAE. The total number of patches, as well as other configurations to train the DAE, were the same as those in the training of the patient-specific DAE for a fair comparison.

#### 2.4.2. Real patient data study

To further demonstrated the effectiveness of the proposed patient-specific manifold scheme, under the approval of the Institutional Review Board, we studied its performance in three real patient cases with liver cancer treated at our institution. We followed dynamic liver contrast enhancement MRI protocol using a 3D T1W mDixon fast field echo sequence under breath hold. The parameters for this sequence were TR 5.3 ms, TE 1.73 ms and 3.6 ms, and flip angle 15 degree. Contrast enhanced MR images were acquired at 90 sec and 180 sec post contrast injection. The one at 90 sec was used as prior image to construct the patient-specific manifold that was then utilized to reconstruct the image at 180 sec. The prior image and the image to be reconstructed differ in image intensity due to contrast enhancement, as well as in structure due to bowel movement and respiratory motion. We studied different levels of noise and undersampling levels. We compared the reconstruction results from our method with those in the clinical standard FT method, the CS method, and the patient-generic manifold method. The resulting image quality was quantitatively assessed using SSIM and PSNR.

#### 2.4.3. Experimental study

We also evaluated the proposed algorithm in an experiment mimicking the clinical practice using a 0.3 T small animal MRI scanner in our lab with a kiwi fruit as the phantom. Specifically, we first scanned the kiwi to generate the prior MR image with 512*times*512 pixels and pixel size of 0.5 mm. The scanning protocol was a Cartesian T2W Rapid Acquisition with Relaxation Enhancement (RARE) (51) sequence with TR 5000 ms, TE 75 ms, echo train length 32, and number of excitation averages 64. The scan was reconstructed with the BM3D denoising filter to remove noise in order to generate a high-quality prior image (52). We extracted one image patch for each pixel and trained the DAE to represent the manifold.

**Figure 4:**
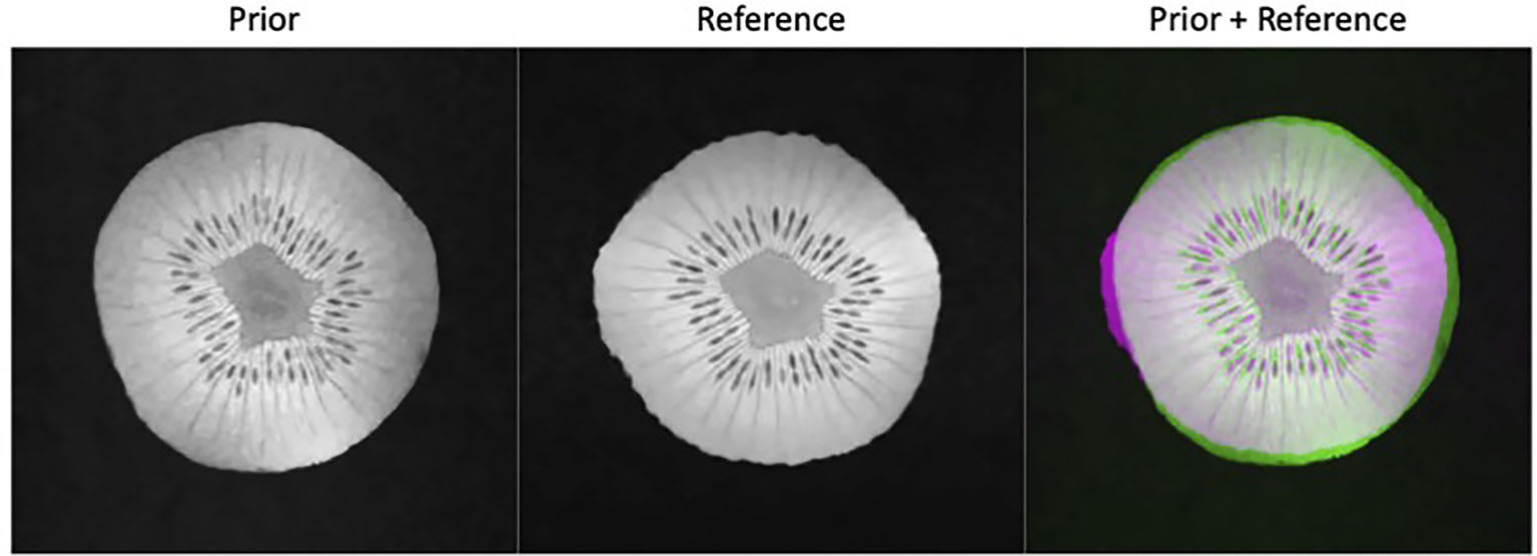
Prior kiwi image (left), the reference image for testing (middle), and the overlay of the prior (green) and reference (magenta) illustrating the deformations (right).

A week later after the first scan, we performed another scan on the same kiwi using the same pulse sequence parameters together with BM3D filter to acquire a high-quality reference ground truth image. The appearance of the kiwi was different from that of the first scan, resulting in a distinct shape (see **Figure 4**). The MRI scanner was then programmed to carry out scans with different undersampling ratios and repetitions of data acquisitions. We reconstructed the MR image using the proposed algorithm. Similarly, to the simulation study, we compared the performance of the proposed method with that of the FT method and the CS method. As the kiwi study does not have a dataset of MR images from multiple ‘patients’, we did not conduct comparison with patient-generic manifold method.

## 3 Results

### 3.1. Results of simulation study

#### 3.1.1. Network training

**Figure 5:**
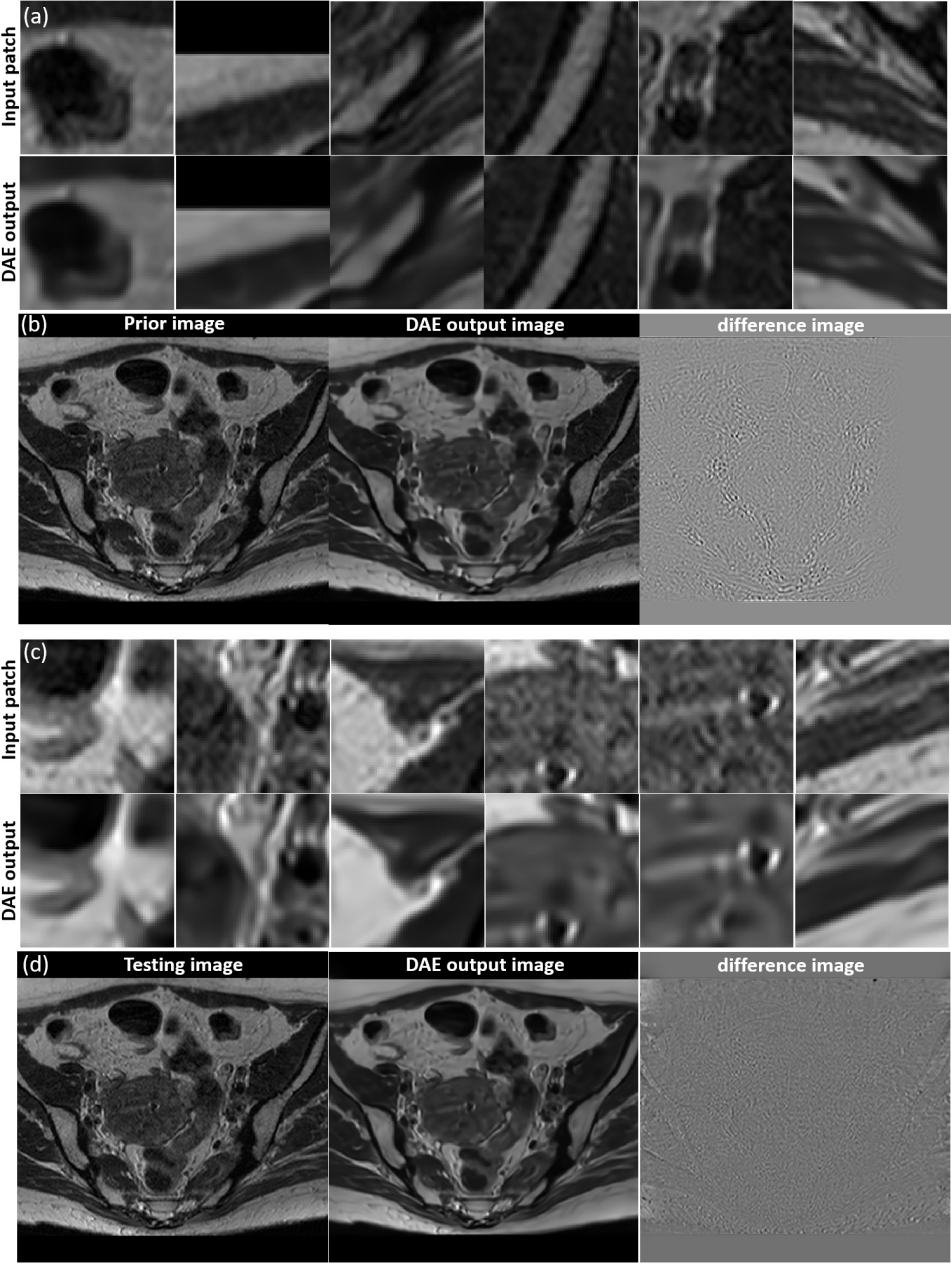
**(A)** Examples of input patches from the prior MR image and their corresponding outputs from the DAE. **(B)** The prior image, image formed by the DAE output patches, and their difference. **(C)** Examples of input patches from the test MR image and their corresponding outputs from the DAE. **(D)** The test MR image, the image assembled by patches from the DAE output, and their difference. All the MR patches and images are displayed in a window of [0, 1], while the difference images are displayed in a window of [-1,1].

We investigated the performance of the DAE established for the specific patient involved in this study, since its quality controls the reconstructed image quality. As such, we first compared the output patches from the DAE with its input of patches from the prior MR image. It was found that the DAE was able to accurately restore the input image patches, achieving an average PSNR of 34.42 (see some examples in **Figure 5A**. We also combined all the output patches from DAE into a complete image and compared it directly to the original prior MR image, see **Figure 5B**. The results show good agreement between the two. We also fed the established DAE with image patches from the test MR image. The resulting patches and the combined image are shown in **Figures 5C, D**, respectively. The results show that DAE fully respected the deformed anatomy and was able to accurately recover image patches for the test image, achieving an average PNSR of 33.55, illustrating the high quality of the patient-specific DAE model.

#### 3.1.2. Image reconstruction

**Figure 6:**
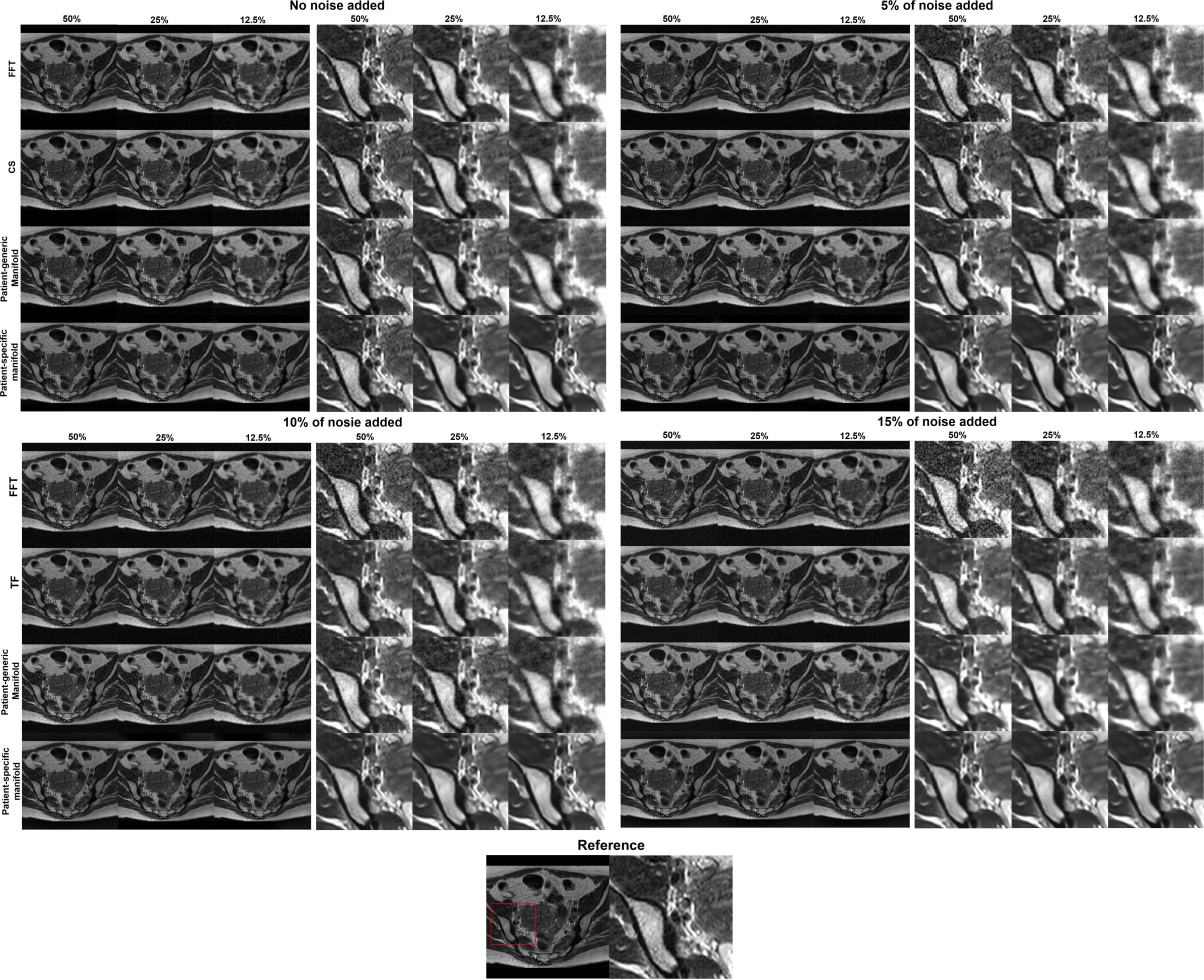
Reconstruction results of the simulation case using data with different undersampling ratios and noise levels. The complete images are displayed in a window of [0,1] and the zoomed-in patches are displayed in a narrower window of [0, 0.6].

In **Figure 6** we present the reconstruction results of different undersampling ratios with different levels of noise added to the k-space data. We compared the reconstruction results of the proposed method with the conventional FT reconstruction, an CS reconstruction method, and the patient-generic manifold prior method. For the FT and CS methods, the image quality degrades severely with less k-space data or increased noise levels. As the undersampling ratio was increased, the resulting images gradually lose fine structures. Noise in the images was increased by the noise in the k-space data. The advantages of the CS method over the FT method became more obvious in the cases with low sampling ratios and high noise levels. The patient-generic manifold algorithm was able to improve the image quality compared to the direct FT reconstruction method, but its performance was comparable to the CS method in most cases. The proposed patient-specific manifold algorithm, on the other hand, obviously obtained superior reconstruction quality compared to all the other three methods. It was able to provide reasonable reconstruction results even with 15% of added noise and up to an undersampling ratio of 12.5%.

This observation was supported by the quantitative comparisons presented in **Table 1**. In all but one cases, the proposed method using the patient-specific prior achieved the best SSIM and PSNR. Only in the case with the least amount of undersampling (50%) without added noise did the FT method have the highest PSNR. In this relatively easy case, the performance of all methods was similar and high, as indicated by the similar SSIM and PSNR values among their results. The slight improvement of SSIM by 0.002 in this case is not expected to be significant. The advantages of our proposed method appeared more significant with larger undersampling rates and more noise contamination, as indicated in both the quantitative evaluation in **Table 1** and by visual assessment in **Figure 6**.

**Table 1:** Quantitative comparisons of FT, CS, patient-generic manifold, and patient-specific manifold methods with different undersampling ratios and noise levels for three liver cases. Each number is the mean value **±** standard deviation. The p-values between the proposed PS-Manifold and each method are included. The best result in each case and statistically significant p-values are highlighted with bold font. FT: Fourier Transform. CS: Compressive Sensing. PG-Manifold: Patient-generic manifold. PS-Manifold: Patient-specific manifold.

Under-sampling		No noise added				10% noise			
	Method	SSIM	*p*	PSNR	*p*	SSIM	*p*	PSNR	*p*
50%	FT	**1.00±0.00**	NA	**58.19±0.78**	**0.00**	0.86**±**0.02	**0.02**	38.78**±**0.60	**0.05**
	CS	**1.00±0.00**	NA	57.51**±**1.09	**0.04**	0.89**±**0.02	**0.04**	36.29**±**0.95	**0.03**
	PG-Manifold	**1.00±0.00**	NA	40.02**±**1.02	**0.00**	0.93**±**0.01	0.18	37.25**±**0.68	0.13
	PS-Manifold	**1.00±0.00**		40.87**±**1.01		**0.95±0.00**		**39.16±0.96**	
25%	FT	**0.98±0.00**	NA	40.19**±**0.66	0.12	0.85**±**0.02	**0.00**	36.46**±**0.89	0.17
	CS	**0.98±0.00**	NA	**41.29±0.67**	0.12	0.89**±**0.02	0.08	36.05**±**0.72	0.44
	PG-Manifold	**0.98±0.00**	NA	37.48**±**1.10	0.33	0.91**±**0.02	0.12	36.14**±**0.44	0.25
	PS-Manifold	**0.98±0.00**		38.79**±**0.81		**0.94±0.00**		**37.01±0.90**	
12.5%	FT	0.93**±**0.01	0.18	32.90**±**1.14	0.59	0.80**±**0.00	**0.00**	32.14**±**0.96	0.16
	CS	0.94**±**0.02	0.69	32.75**±**0.48	0.39	0.87**±**0.02	0.08	32.35**±**0.18	0.22
	PG-Manifold	0.93**±**0.01	0.12	32.57**±**0.93	0.20	0.88**±**0.03	0.25	32.29**±**0.80	0.12
	PS-Manifold	**0.95±0.00**		**33.22±0.45**		**0.92±0.01**		**32.95±0.43**	

Under-sampling		20% noise				30% noise			
	Method	SSIM	*p*	PSNR	*p*	SSIM	*p*	PSNR	*p*
50%	FT	0.70**±**0.03	**0.00**	32.77**±**0.61	**0.00**	0.60**±**0.00	**0.01**	29.27**±**0.60	**0.00**
	CS	0.80**±**0.04	**0.02**	34.19**±**0.10	**0.03**	0.67**±**0.04	**0.04**	31.30**±**1.15	**0.01**
	PG-Manifold	0.82**±**0.03	**0.03**	34.14**±**0.41	**0.00**	0.69**±**0.03	0.09	31.20**±**0.34	**0.04**
	PS-Manifold	**0.88±0.02**		**35.29±0.45**		**0.81±0.03**		**32.85±0.96**	
25%	FT	0.70**±**0.03	**0.01**	32.58**±**0.61	**0.03**	0.61**±**0.04	**0.01**	29.37**±**0.70	**0.00**
	CS	0.75**±**0.06	**0.03**	33.40**±**0.96	0.12	0.62**±**0.05	**0.04**	30.70**±**0.83	**0.02**
	PG-Manifold	0.79**±**0.17	0.18	33.75**±**0.35	0.27	0.68**±**0.05	0.14	30.91**±**0.31	0.09
	PS-Manifold	**0.87±0.02**		**35.02±0.73**		**0.79±0.01**		**32.51±0.99**	
12.5%	FT	0.66**±**0.03	**0.01**	30.38**±**0.68	**0.02**	0.58**±**0.04	**0.04**	28.44**±**0.53	**0.00**
	CS	0.72**±**0.05	**0.04**	30.89**±**0.66	**0.02**	0.62**±**0.02	0.09	29.33**±**0.31	**0.03**
	PG-Manifold	0.74**±**0.03	0.07	31.36**±**0.62	**0.04**	0.65**±**0.05	0.07	29.62**±**0.44	**0.01**
	PS-Manifold	**0.83±0.01**		**31.98±0.44**		**0.77±0.07**		**30.75±0.51**	

Each number is the mean value ± standard deviation. The p-values between the proposed PS-Manifold and each method are included. The best result in each case and statistically significant p-values are highlighted with bold font. FT: Fourier Transform. CS: Compressive Sensing. PG-Manifold: Patient-generic manifold. PS-Manifold: Patient-specific manifold.

**Figure 7:**
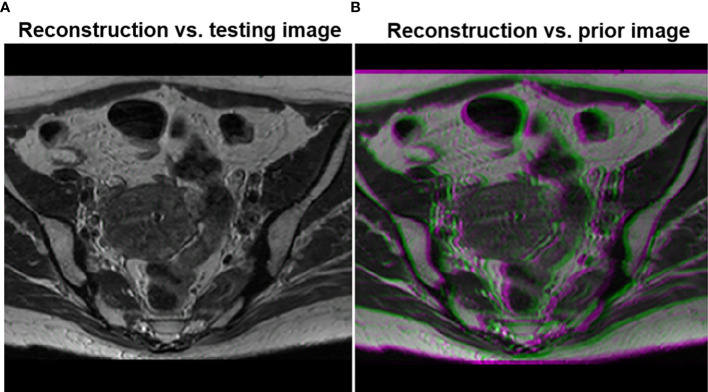
**(A)** Overlay of the testing image and the reconstructed image using the proposed method. **(B)** Overlay of the reconstructed image and the prior image.

To demonstrate that the anatomical structure of the reconstruction result was not biased by the prior image, we overlaid the image reconstructed by the proposed method with the ground truth testing image in **Figure 7**. The anatomical structures in the two images were visually indistinguishable, as indicated by the sharp edges of the overlaid image. In contrast, when we overlaid the reconstructed image on the prior image, the blurry result demonstrated that the reconstructed image had a different anatomical structure, representing the deformation between the prior image and the test image.

### 3.2. Results of real patient data study

**Figure 8:**
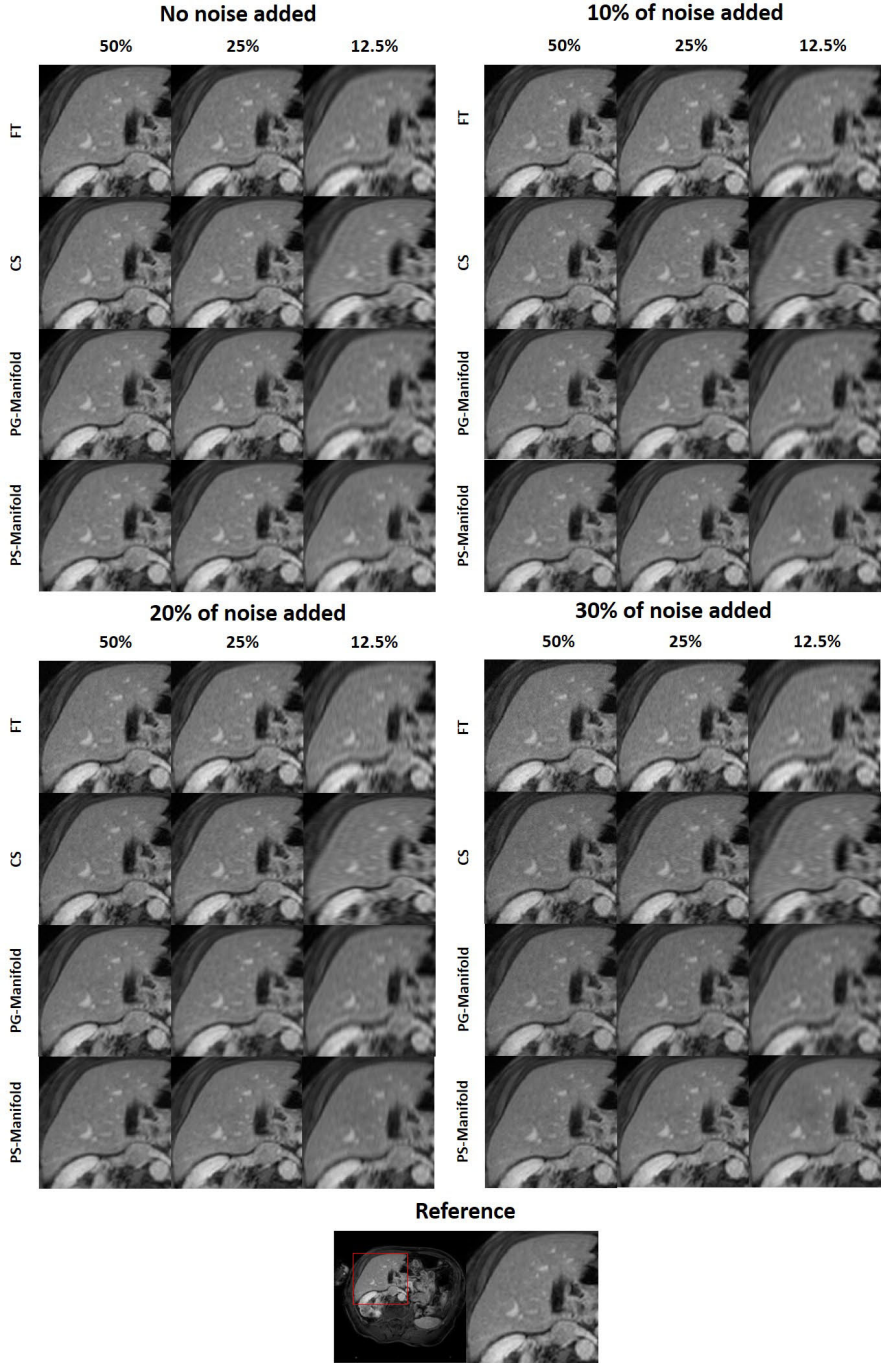
Reconstruction results of a patient case using data with different undersampling ratios and noise levels. The complete reference image and the zoomed-in patches are displayed in a window of [0, 1].

The reconstruction results of one liver case with different undersampling ratios and different levels of noise are shown in **Figure 8**. The advantages of the patient-specific algorithm in terms of image quality became more obvious at more challenging cases with high noise levels and undersampling ratios. Paired t-tests were performed to test if the patient-specific manifold was better than each of the other methods. *P* values less than 0.05 were considered significant. The comparisons between the proposed method and each of the other three methods are presented in **Table 2**. Similar findings were observed to the previous simulation case. Overall, the proposed patient-specific manifold algorithm outperformed others in terms of reconstructing images with higher SSIM and PSNR values. The advantage was especially prominent in the cases with the larger amount of undersampling and noise. Generally, the statistical significance became more obvious in more challenging cases.

**Table 2:** Quantitative comparisons of FT, CS, patient-generic manifold, and patient-specific manifold methods with different undersampling ratios and noise levels in the simulation case.

Under-sampling		No noise added		5% noise		10% noise		15% noise	
	Methods	SSIM	PSNR	SSIM	PSNR	SSIM	PSNR	SSIM	PSNR
50%	FT	0.949	**43.48**	0.660	29.83	0.540	25.68	0.400	21.23
	CS	0.949	43.38	0.762	33.62	0.737	28.88	0.648	23.58
	PG-Manifold	0.947	42.31	0.743	31.77	0.606	26.76	0.581	24.95
	PS-Manifold	**0.951**	43.04	**0.821**	**33.81**	**0.749**	**29.52**	**0.734**	**27.24**
25%	FT	0.799	33.35	0.687	30.31	0.588	24.44	0.499	23.48
	CS	0.798	33.31	0.729	31.17	0.703	25.42	0.673	25.11
	PG-Manifold	0.797	33.19	0.719	30.69	0.614	25.91	0.550	25.19
	PS-Manifold	**0.852**	**33.94**	**0.862**	**32.04**	**0.816**	**29.04**	**0.735**	**28.21**
12.5%	FT	0.676	28.33	0.629	27.68	0.563	22.60	0.503	22.28
	CS	0.676	28.32	0.648	27.82	0.627	22.92	0.565	22.65
	PG-Manifold	0.674	28.29	0.640	27.72	0.587	23.48	0.591	22.70
	PS-Manifold	**0.862**	**31.62**	**0.853**	**31.18**	**0.784**	**28.25**	**0.727**	**27.26**

The best result in each case is highlighted with bold font. FT: Fourier Transform. CS: Compressive Sensing. PG-Manifold: Patient-generic manifold. PS-Manifold: Patient-specific manifold.

**Figure 9:**
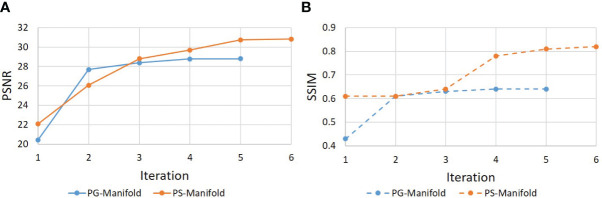
Evolution of (a) PSNR and (b) SSIM during the iterative reconstruction process for a real patient case.

**Figure 9** shows the evolution of PSNR and SSIM along the iteration process for an example of a real patient case. The curves saturated at the end, indicating convergence of the method. The patient-specific manifold reconstruction achieved better PSNR and SSIM than the patient-generic manifold. We also remark that the convergence behavior was only numerically demonstrated, but we did not have a theoretical justification for the reconstruction process involving complex operations by the DAE.

### 3.3 Results of experental study

#### 3.3.1 Network training

**Figure 10:**
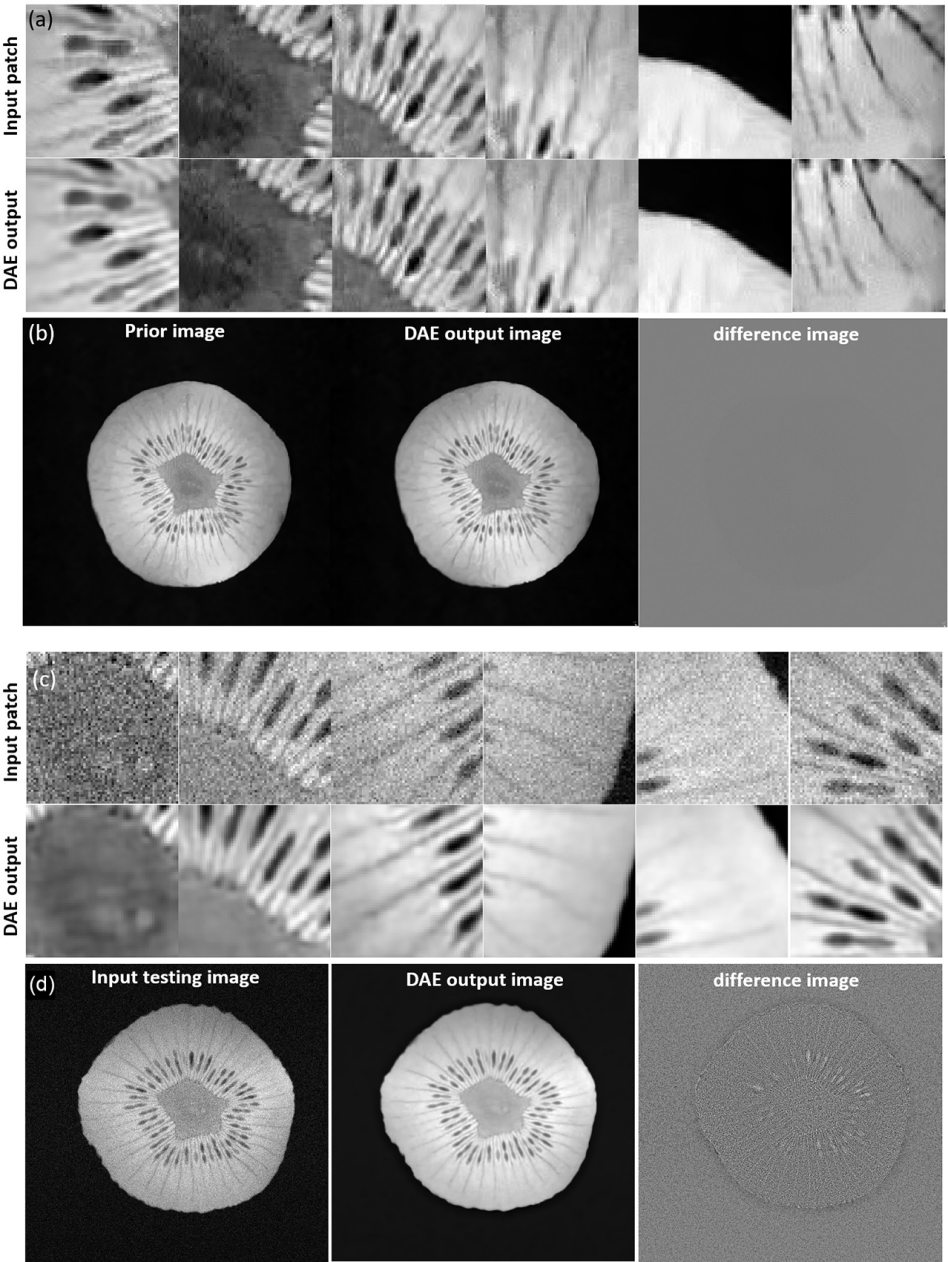
**(A)** Input and output patches of the DAE for the prior kiwi image. **(B)** Prior Kiwi image, the image assembled by patches from DAE output, and their difference image. **(C)** Input and output patches of the DAE for a testing kiwi image. **(D)** A testing Kiwi image, the image assembled by patches from DAE output, and their difference images. All the MR patches and images are displayed in a window of [0, 1], while the difference images are displayed in a window of [-1, 1].

We first investigated the performance of the DAE trained using the prior kiwi image. After feeding in patches of the prior kiwi images as the input, the trained DAE was able to accurately recover the input patches with an average PSNR of 37.86. In **Figures 10A, B**, we show several example patches and the combined kiwi image recovered by the established DAE, respectively. With its effectiveness confirmed on the prior image, we further evaluated the performance of the trained DAE on the testing data acquired for the same kiwi several days after the acquisition of the prior image. The recovering results for the testing image patches can be found in **Figures 10C, D**. Note that the input image was reconstructed using the direct FT method. Although we have used the fully sampled data with the largest number of repetitions available in our experimental dataset, a relatively high level of noise still existed in the reconstructed image. Through visually examining the patches processed by the DAE and the completed image shown in **Figures 10C, D**, we found that the established DAE was able to recover most of the structures in the input image while substantially suppressing the noise contamination.

#### 3.3.2. Image reconstruction

**Figure 11:**
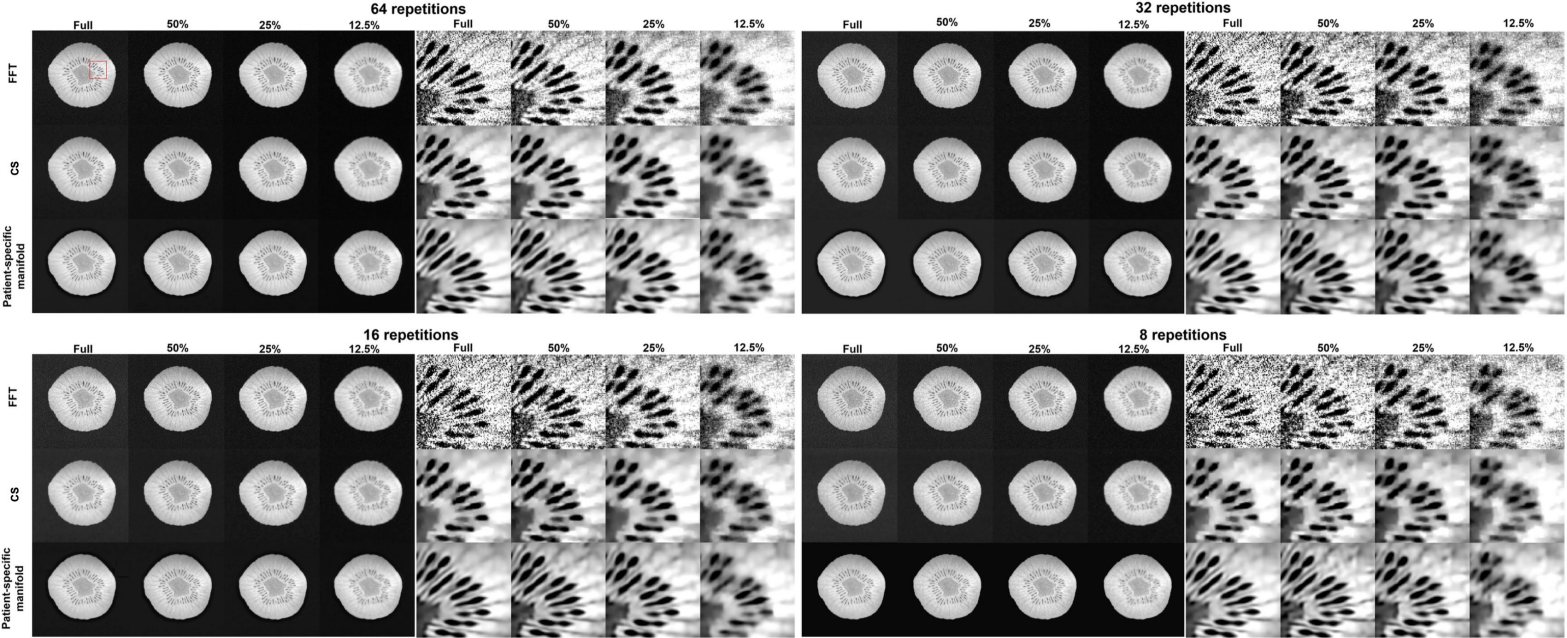
Reconstruction results of the kiwi using data with different undersampling ratios and with varying noise levels by adjusting the number of scan repetitions. These are reconstructed using a Fast Fourier Transfer (FFT), a tight-frame compressed sensing method (CS), and the proposed patient-specific manifold. The complete images are displayed in a window of [0, 1] and the zoomed-in patches are displayed in a narrower window of [0.65, 0.95].

In **Figure 11**, we present the reconstruction results with different undersampling ratios and number of data acquisition repetitions. Note that less repetitions improves the acquisition efficiency, while the noise level is unavoidably amplified due to less averaging. Among the three methods, the proposed method visually provided the best reconstruction image quality in all testing scenarios. Overall, the direct FT method suffered the most from noise. The undersampling process also may degrade its reconstruction quality with loss in detailed textures, but at the same time reduce noise to a certain extent. Compared to the direct FT method, the CS method was effective in terms of improving the reconstruction quality in most cases by suppressing noise. However, the images were blurred in the noise removal process. The advantage of the proposed patient-specific manifold algorithm over the direct FT method and the CS method can be easily observed in **Table 3**. In the most challenging case with 12.5% undersampling and 8 repetitions, the SSIM was increased from 0.772 in the FT method to 0.853 in our method, and the PSNR from 26.80 to 28.40.

**Table 3:** Quantitative comparisons of FT, CS, and patient-specific manifold methods with different undersampling ratios and noise levels in the experimental case. .

Under-sampling		64 repetitions		32 repetitions		16 repetitions		8 repetitions	
	Methods	SSIM	PSNR	SSIM	PSNR	SSIM	PSNR	SSIM	PSNR
Full	FT	0.747	24.46	0.545	19.77	0.482	17.73	0.421	16.72
	CS	0.807	29.44	0.783	25.35	0.788	25.32	0.739	21.21
	PS-Manifold	**0.958**	**35.59**	**0.925**	**31.42**	**0.892**	**28.86**	**0.748**	**22.27**
50%	FT	0.852	30.06	0.729	24.99	0.661	22.69	0.584	21.45
	CS	0.818	30.31	0.720	27.94	0.780	27.85	0.693	26.09
	PS-Manifold	**0.911**	**32.79**	**0.944**	**32.28**	**0.935**	**31.69**	**0.919**	**30.74**
25%	FT	0.850	30.50	0.806	27.90	0.762	26.24	0.716	23.42
	CS	0.785	29.32	0.788	28.67	0.822	28.62	0.796	25.97
	PS-Manifold	**0.922**	**31.08**	**0.947**	**31.77**	**0.894**	**30.50**	**0.918**	**30.57**
12.5%	FT	0.799	**27.73**	0.812	27.94	0.806	27.15	0.772	26.80
	CS	**0.804**	27.70	0.780	27.64	0.802	27.79	0.786	27.20
	PS-Manifold	0.735	27.51	**0.920**	**29.72**	**0.811**	**27.99**	**0.853**	**28.40**

The best result in each case is highlighted with bold font. FT: Fourier Transform. CS: Compressed Sensing. PS-Manifold: Patient-specific manifold.

## 4 Discussion

It is generally understood that a DNN, as a universal function approximator, can be trained to represent an unknown highly complex function. In this study, we employed a DAE to represent the manifold of MR images. As mentioned in Section 1, the effectiveness of the manifold-constrained image reconstruction lies in how well the manifold can be developed to represent the image to be reconstructed, so as to effectively provide information to compensate the information loss caused by data undersampling and amplified noise. This study innovatively noticed that the specific clinical context of MRgRT offers the opportunity of using the patient-specific prior MR information. Using the patient-specific prior MR image to train the DAE allows a focused development of the manifold that is known to be valid for the patient of interest. Hence, this ensures the effectiveness of the manifold for the MR image reconstruction problem of this particular patient.

Another advantage of the reconstruction algorithm is interpretability. While the trained DNN still maintained a black-box nature by itself, its role can be generally understood as improving image quality by mapping an image outside the manifold to the one on the manifold. As the manifold was trained to represent the specific patient’s image feature, this operation enforces relevance of the mapped image to the clinical context. Furthermore, the DNN was incorporated into a reconstruction framework with a geographically interpretable workflow (**Figure 1A**). These interpretations are expected to be of critical importance for us to establish trust to the algorithm and to facilitate its clinical applications (53).

In principle, if the patient-generic manifold can be trained to represent all patients’ prior images, it should already contain the patient-specific prior manifold, and thus achieve a non-inferior performance to the approach using the patient-specific prior manifold in the image reconstruction problem. Nonetheless, it may be practically challenging to develop such a manifold. First, it is difficult to assert that the manifold trained on images from a group of patients is valid for the new image to be reconstructed. Second, for the patient-generic approach, the DNN’s capacity has to be large enough to represent image characteristics of a large number of patients, requiring a DNN with a large size and complex structure. Training this kind of DNN with a substantial amount of data raises the concern of computational burden. With these considerations and patient-specific prior information available, we believe it is preferable to use a DNN to specifically target the patient of interest, which not only ensures the validity of this approach, but is also numerically more tractable. We also emphasize that this study trained the DAEs independent of the reconstruction workflow. Only after the training was finished, was the network plugged into the reconstruction algorithm as one key element. The comparison between patient-specific and patient-generic training was performed under this setting. Recently, novel studies demonstrated that training in an end-to-end fashion could further increase the effectiveness of network training and hence resulting image reconstruction (26–28). Therefore, the comparison study between patient-specific and patient-generic trainings should be understood only in the particular setting in this study. Under the training in an end-to-end way, the patient generic method may still achieve better performance.

Overfitting is a general concern for methods employing prior information. From an application perspective, since the intended use of the reconstructed images is for image guidance, avoiding overfitting to the anatomy from the prior image is most critical. Our model was trained to learn the prior information at the patch level, which could be helpful in preventing overfitting in this regard. As demonstrated in our study, the reconstructed image captured the new anatomy and was not obviously biased to the prior anatomy. In the model training stage, we used dropout layers and batch normalization to regularize gradient and reduce overfitting.

In the reconstructed images, a mild degree of blurring was noticed. As the undersampling scheme sampled the low frequency domain, the high frequency information was lost. The resulting images hence appeared generally blurry. The role of the DAE was to provide missing high-frequency information and hence increase sharpness. However, since this high-frequency information was not fully recovered accurately, the residual error manifested as blurriness in the images. We do not expect this to be a major problem for the specific application of image guidance in RT, because the alignment could be performed with reasonable accuracy without needing all detailed structures. Down the road, it is also an important study to investigate the optimal undersampling pattern within this framework for the best trade-off between image guidance accuracy and scan time.

The current study has several limitations. First, as an initial study demonstrating the idea of using patient-specific DAE-manifold to assist MRI reconstruction, for simplicity, we did not consider the phase of the images and assumed that the phase correction step has been performed before reconstruction. This preprocessing step may be achieved *via* existing phase correction methods (54, 55). As such, we assumed the solution is a real-valued image, and enforced this during each iteration step. The performance of this approach was found acceptable in the tests presented in this study. This assumption has often been employed in many studies as an initial step to test new MR reconstruction algorithms [16,8]. Yet in reality, phase errors caused by variations in the resonance frequency, flow, and motion may violate this real-value assumption. For iterative reconstruction algorithms including ours, the algorithm improves quality of the solution based on prior knowledge at each iteration step. This is often done on the magnitude image. Correcting the phase errors helps improving quality of the solution and the utilization of prior information. For this purpose, the iterative algorithms could be extended to include a phase correction step in the iterative process, for example using the classical POCS algorithm (56). However, including the phase correction step will lead to a mathematically complex optimization problem, and the performance of the proposed method may decrease.

Second, evaluation of the study could be further improved. Effectiveness of the proposed method and its advantage over the patient-generic manifold approach were only demonstrated in a limited number of patient simulation cases and a non-patient experimental study. The simple textures in a kiwi may not be sufficient to simulate the organ movements and anatomical change due to RT treatments between the image prior that was used to construct the manifold and the images to be reconstructed. In a real treatment course, the time duration between treatment planning and treatment, and the time between multi-fractionated treatments can allow the patient to undergo anatomy changes. Our study is the initial step proposing the idea using a patient-specific prior to facilitate MR reconstruction in the unique context of MRgRT. Hence, the changes shown in this paper are for the purpose of illustrating the principle. It is ongoing work to evaluate the proposed method on the real patient MR images during a RT course. As for the metrics used, SSIM and PSNR were utilized to characterize image quality in terms of structural and intensity accuracy. Additional metrics could be added, but this may reflect the results and accuracy from similar perspectives. For future evaluations in clinical applications, reader studies may be included.

Third, the reconstruction method is in fact computationally heavy due to the need to process all the patches in an image using the DAE in each iterative step of the Algorithm 1. The complexity of this operation is of the order of the number of pixels. This was caused by the fact that the manifold was built for patches, as opposed to the entire image. A manifold of the latter is desired from this perspective, but is hard to build due to the scarcity of prior images of the specific patient. Potential approaches to overcome this computational challenge include increasing computational power and designing algorithms to process only a subset of all patches. Additionally, as a proof-of-principle study, we only considered a 2D image reconstruction here for the purpose of easy network training and computing. While extending the method to 3D cases is straightforward, e.g. by employing 3D DAE and MR reconstruction algorithms to accommodate clinically relevant 3D MR sequences in MRgRT, this would certainly further increase the computational burden.

## 5 Conclusion

The unique clinical scenario of MRgRT offers high-quality treatment planning MR images of a patient as patient-specific prior information to support the reconstruction of MR images for image guidance purposes. In this paper, we developed an algorithm exploiting the patient-specific image prior to facilitate the reconstruction of MR images with an undersampled data acquisition. We trained a DAE to form a manifold of the prior MR image of the specific patient. The manifold was incorporated in the image reconstruction problem as a regularization term to restore MR images from the undersampled data. Compared with the standard FT-based reconstruction method, a tight frame-based CS method, and a patient-generic manifold method, our method produced reconstructions of improved image quality.

## Data availability statement

The datasets presented in this article are not readily available because The data that support the findings of this study are available from the corresponding author upon reasonable request and subject to institutional IRB approval. Requests to access the datasets should be directed to XJ, xun.jia@utsouthwestern.edu.

## Ethics statement

The studies involving human participants were reviewed and approved by Internal Review Board of UT Southwestern Medical Center. Written informed consent for participation was not required for this study in accordance with the national legislation and the institutional requirements.

## Author contributions

JG and YGa are co-first authors and contributed equally to this study. XJ, CS, and JD contributed to the study concepts and study design. JG, YGa, YGo and CS contributed to data acquisition and image reconstructions. All authors contributed to data analyses and interpretation, manuscript preparation and editing. All authors contributed to the article and approved the submitted version.

## Funding

This work is supported in part by the Cancer Prevention and Research Institute of Texas under Grant RP200573 and National Institutes of Health under Grants R01CA227289, R01CA237269, and R37CA214639.

## Conflict of interest

The authors declare that the research was conducted in the absence of any commercial or financial relationships that could be construed as a potential conflict of interest.

## Publisher’s note

All claims expressed in this article are solely those of the authors and do not necessarily represent those of their affiliated organizations, or those of the publisher, the editors and the reviewers. Any product that may be evaluated in this article, or claim that may be made by its manufacturer, is not guaranteed or endorsed by the publisher.
